# Dextromethorphan Modulates the Osteogenic–Adipogenic Balance in Rat Bone Marrow Mesenchymal Stem Cells

**DOI:** 10.3390/cells15110995

**Published:** 2026-05-28

**Authors:** Jian-Hong Lin, Yu-Po Luo, Pei-Ching Ting, Min-Pei Ko, Kun-Ta Yang

**Affiliations:** 1 Division of Experimental Surgery, Department of Surgery, Hualien Tzu Chi Hospital, Buddhist Tzu Chi Medical Foundation, No. 707, Sec. 3, Zhongyang Rd., Hualien 97002, Taiwan; 101327102@gms.tcu.edu.tw; 2 Master Program in Biomedical Sciences, School of Medicine, Tzu Chi University, No. 701, Sec. 3, Zhongyang Rd., Hualien 97004, Taiwan; qwe78945662@gmail.com (Y.-P.L.); 112333110@gms.tcu.edu.tw (M.-P.K.); 3 PhD Program in Pharmacology and Toxicology, School of Medicine, Tzu Chi University, No. 701, Sec. 3, Zhongyang Rd., Hualien 97004, Taiwan; 110752101@gms.tcu.edu.tw; 4 Department of Surgery, Hualien Tzu Chi Hospital, Buddhist Tzu Chi Medical Foundation, No. 707, Sec. 3, Zhongyang Rd., Hualien 97002, Taiwan; 5 Department of Physiology, School of Medicine, Tzu Chi University, No. 701, Sec. 3, Zhongyang Rd., Hualien 97004, Taiwan

**Keywords:** dextromethorphan, mesenchymal stem cells, adipogenic differentiation, osteogenic differentiation

## Abstract

Bone marrow-derived mesenchymal stem cells (BMSCs) maintain skeletal homeostasis by balancing adipogenic and osteogenic differentiation, yet clinically used drugs that bias this fate choice and their mechanisms remain incompletely defined. Here, we investigated whether dextromethorphan (DXM), a widely used antitussive, modulated lineage commitment in rat BMSCs and interrogated candidate upstream signaling modules. Rat BMSCs were induced with adipogenic medium or osteogenic medium in the presence of DXM (30 μM). Adipogenesis and osteogenesis were quantified using Oil Red O and Alizarin Red S staining with elution-based quantification, and lineage markers were measured by RT-qPCR. Intracellular Ca^2+^ and ROS were analyzed using flow cytometry, and the levels of p-AKT and p-ERK were assessed through Western blotting analysis. Under adipogenic induction, DXM increased lipid droplet accumulation and the mRNA levels of Pparγ and Fabp4. Although DXM elevated Ca^2+^ and ROS, the chelation of intracellular Ca^2+^ and pharmacological inhibition of Sig-1R/PLC–IP3R signaling, redox/ROS, NMDA receptors, AKT/ERK, Kv channels, bitter taste receptor-related signaling, and mTOR did not attenuate the DXM-enhanced adipogenesis. DXM reduced p-ERK without increasing p-AKT; U0126 lowered basal adipogenesis but did not block the DXM effect. Under osteogenic induction, DXM reduced matrix mineralization and downregulated *Runx2* and *Bglap* mRNA levels, while *Wwtr1* mRNA levels were not significantly changed. DXM also partially reversed the osteogenic induction-associated reduction in *Mtor* mRNA. Separately, under adipogenic induction, rapamycin attenuated baseline adipogenesis but did not prevent the additional lipid accumulation induced by DXM. Collectively, DXM shifted the osteogenic–adipogenic balance toward adipogenesis through a non-canonical mechanism.

## 1. Introduction

Bone marrow-derived mesenchymal stem cells (BMSCs) exhibit multilineage potential and can differentiate into adipocytes or osteoblasts in response to specific microenvironmental signals. The balance between adipogenic and osteogenic differentiation is essential for skeletal homeostasis; its disruption is linked to osteoporosis, age-related bone loss, and metabolic abnormalities, and is often accompanied by increased bone marrow adiposity and reduced osteoblastogenesis [1,2]. At the molecular level, lineage commitment is governed by coordinated transcriptional programs, including the upregulation of adipogenic regulators such as peroxisome proliferator-activated receptor gamma (PPARγ) and downregulation of osteogenic transcription factors such as runt-related transcription factor 2 (Runx2), reflecting reciprocal antagonism between adipogenic and osteogenic gene networks [3,4]. Identifying clinically used agents that modulate this fate balance and defining their molecular mechanisms remain highly relevant to regenerative medicine and metabolic bone disease [5,6].

Dextromethorphan (DXM), a widely used antitussive, has been reported in neuronal and glial preparations to attenuate NADPH oxidase (NOX)-linked oxidative stress and to modulate Ca^2+^ handling [7,8]. DXM has also been linked to membrane-associated targets, including N-methyl-D-aspartate receptors (NMDARs), the sigma-1 receptor (Sig-1R), and extra-oral bitter taste receptors (TAS2Rs), suggesting potential to intersect signaling networks relevant to cell fate regulation [9,10,11]. In osteoblast-lineage systems, DXM suppresses mineralization, alkaline phosphatase activity, and osteogenic marker expression in MC3T3-E1 cells, with in vivo evidence from zebrafish larvae and rat calvarial defects supporting impaired ossification and reduced bone regeneration under the conditions tested [12]. In addition, the DXM metabolite 3-hydroxymorphinan promotes mitochondrial biogenesis and adipocyte browning through the AMP-activated protein kinase signaling in 3T3-L1 adipocytes [13]. Collectively, these findings suggest that DXM may influence the osteogenic–adipogenic balance; however, whether DXM and/or its bioactive metabolites directly bias BMSC lineage outcomes and which upstream signaling modules mediate this effect remain insufficiently defined.

BMSC fate decisions are regulated by interconnected signaling modules that converge on lineage-specific transcriptional and metabolic programs [5,14]. Calcium-dependent signaling and reactive oxygen species (ROS) pathways can shape differentiation by modulating mitochondrial function and redox-sensitive transcription [15,16]. Kinase cascades, including protein kinase B (AKT), extracellular signal-regulated kinase (ERK), and mechanistic target of rapamycin (mTOR) pathways, are frequently implicated in adipogenic versus osteogenic outcomes in a context-dependent manner [17,18]. In addition, membrane-associated inputs, including NMDAR-related mechanisms, Sig-1R-associated processes, and TAS2Rs, can couple extracellular cues to intracellular signaling networks that shape lineage programs [19,20,21]. Additional second-messenger and membrane pathways, including phospholipase C (PLC)-dependent inositol 1,4,5-trisphosphate (IP3) generation, downstream IP3 receptor (IP3R)-associated Ca^2+^ signaling (PLC-IP3-IP3R) [22,23], and ion channel activity [24,25] that can engage PLC-IP3-Ca^2+^ transduction [21,26], may further modulate intracellular states that influence differentiation. At the transcriptional level, co-regulators such as transcriptional co-activator with PDZ-binding motif (TAZ encoded by Wwtr1) participate in controlling the adipogenic–osteogenic balance [27]. This interdependence and context sensitivity underscore the need for systematic pathway interrogation when the mechanistic basis of lineage-modulating compounds is defined [5,14].

In the present study, we investigated whether DXM altered lineage outcomes in rat BMSCs and interrogated candidate upstream signaling modules that may contribute to this effect. We showed that DXM enhanced adipogenic differentiation, reflected by increased lipid accumulation and upregulation of *Pparγ* and *Fabp4* mRNA levels, while osteogenic differentiation is concurrently suppressed, indicated by reduced matrix mineralization and the downregulation of *Runx2* and *Bglap* mRNA levels. Systematic pharmacological and transcriptional interrogation suggest that the incremental pro-adipogenic effect of DXM is not predominantly mediated by Ca^2+^-associated signaling including Sig-1R and PLC-IP3-IP3R modules, ROS signaling, NMDAR-related inputs, AKT/ERK-associated cascades, voltage-gated potassium (Kv) channel, TAS2Rs, or *Mtor* and *Wwtr1* mRNA levels under our experimental conditions. Collectively, the findings of the present study suggest that DXM shifted the osteogenic–adipogenic balance in rat BMSCs through a non-canonical regulatory mechanism.

## 2. Materials and Methods

### 2.1. Chemicals

Dexamethasone, L-ascorbate-2-phosphate, indomethacin, β-glycerophosphate, triethyl phosphate, acetic acid, ammonium hydroxide, L-glutamate, 4-aminopyridine (4-AP), gamma-aminobutyric acid (GABA), NMDA, MK-801, Oil Red O, and DXM were purchased from Sigma-Aldrich (St. Louis, MO, USA). Alizarin Red S, rapamycin, U0126, and U73122 were obtained from Merck Millipore (Burlington, MA, USA). MnTBAP was purchased from Calbiochem (San Diego, CA, USA), while BD1063 and BD1047 were sourced from Abcam (Cambridge, UK). 1,2-bis (2-aminophenoxy)ethane-N,N,N′,N′-tetraacetic acid tetra (acetoxymethyl ester) (BAPTA-AM) was purchased from Adooq Bioscience (Irvine, CA, USA). DXM was dissolved in double-distilled water.

### 2.2. Preparation and Expansion of Rat BMSCs

BMSCs were harvested from femurs and tibias of urethane-anesthetized (1.5 g/kg, i.p.) adult male rats (300–420 g) following previously established protocols [28] and flushed with PBS containing 1% penicillin/streptomycin (Gibco; Grand Island, NY, USA). The procedure was approved by the Institutional Animal Care and Use Committee of Tzu Chi University (IACUC Approval No. 107038; approval date: 23 July 2018). The suspension was filtered (100 μm) and mononuclear cells were isolated by Ficoll-Paque PLUS (GE Healthcare; Uppsala, Sweden) density gradient centrifugation, followed by PBS washes. Cells were cultured in α-MEM (Gibco) supplemented with 15% FBS (Gibco) and 1% penicillin/streptomycin at 37 °C with 5% CO_2_, with medium changes every 2 days. Cells from passages 2–5 were used for experiments.

### 2.3. Adipogenic Differentiation

Rat BMSCs were plated onto poly-L-lysine-coated glass coverslips (10 μg/mL; 24 mm diameter) at 2 × 10^4^ cells/cm^2^. Adipogenic differentiation was initiated by switching to adipogenic medium (AM), consisting of DMEM–low glucose (Gibco) supplemented with 10^−7^ M dexamethasone, 10 μg/mL indomethacin, and 50 μg/mL L-ascorbic acid 2-phosphate. Cells were induced for 10–13 days with medium replacement every 2 days; the induction duration was defined by the appearance of refractive intracellular lipid droplets to ensure comparable differentiation across groups. At each medium change, AM containing DXM was freshly prepared from light-protected stock solutions and replaced to replenish DXM and minimize potential degradation.

### 2.4. Osteogenic Differentiation

Rat BMSCs were seeded in 24-well plates at 2 × 10^4^ cells/cm^2^. Osteogenesis was induced by replacing the culture medium with osteogenic medium (OM), consisting of α-MEM supplemented with 10^−8^ M dexamethasone, 10 mM β-glycerophosphate, and 50 μg/mL L-ascorbic acid 2-phosphate. Osteogenic induction was continued for 21–24 days with medium changes every 2 days; the induction duration was determined by the formation of mineralized nodules to ensure consistent differentiation across groups. At each medium change, OM containing DXM was freshly prepared from light-protected stock solutions and replaced to replenish DXM and minimize potential degradation.

### 2.5. Oil Red O Staining

Adipogenic differentiation was evaluated by Oil Red O staining. Cells were fixed with 10% formalin for 30 min, stained for 10 min with a filtered Oil Red O working solution (in 60% triethyl phosphate), and imaged using an inverted microscope. For quantification, stained cultures were air-dried, and Oil Red O was eluted with 60% triethyl phosphate for 10 min. Absorbance was measured at 490 nm.

### 2.6. Alizarin Red S Staining

Matrix mineralization was assessed using Alizarin Red S staining (40 mM, pH 4.1). Cells were fixed with 10% formalin for 15 min, stained, and imaged. For quantification, bound dye was extracted by incubating the stained cultures in 10% acetic acid for 30 min, followed by heating at 85 °C. After centrifugation, the supernatant was neutralized with 10% ammonium hydroxide and read at 405 nm using a microplate reader.

### 2.7. Intracellular Ca^2+^ Analysis

Intracellular Ca^2+^ was measured through Fluo-3/AM staining. Rat BMSCs were incubated with 2.5 μM Fluo-3/AM (Invitrogen; Carlsbad, CA, USA) for 30 min at room temperature in the dark, and fluorescence was analyzed using a Gallios Flow Cytometer (Beckman Coulter, Brea, CA, USA) (Ex/Em: 488/525 nm).

### 2.8. Intracellular ROS Analysis

Intracellular ROS levels were assessed using CM-H2DCFDA. Cells were incubated with 2.5 μM CM-H2DCFDA (Molecular Probes, Eugene, OR, USA) for 30 min at room temperature in the dark, and fluorescence was measured by Gallios flow cytometry (Ex/Em: 488/525 nm).

### 2.9. Quantitative Real-Time PCR

Total RNA was extracted using TRIzol reagent (Ambion; Carlsbad, CA, USA). cDNA was synthesized from 3 μg total RNA using the Verso cDNA Kit (Thermo, Waltham, MA, USA). qPCR was performed using Maxima SYBR Green qPCR Master Mix (2×) with ROX (Thermo) on a LightCycler 480 System (Roche; Basel, Switzerland). Primer sequences were as follows: *Pparγ*, forward: 5′-TTG AGT GCC GAG TCT GTG GGG ATA A-3′, reverse: 5′-CAG GGA GGC CAG CAT CGT GTA GA-3′; *Fabp4*, forward: 5′-GTT GGC TTC GCC ACC AGG AAA GT-3′, reverse: 5′-AGT ACT CTC TGA CCG GAT GAC GA-3′; *Runx2*, forward: 5′-CTC CAA CCC ACG AAT GCA CTA-3′, reverse: 5′-GTG AGT GGT GGC GGA CAT G-3′; *Bglap*, forward: 5′-GCC CTG ACT GCA TTC TGC CTC T-3′, reverse: 5′-TCA CCA CCT TAC TGC CCT CCT G-3′; *Wwtr1*, forward: 5′-AGG ATC AGG ATG CGT CAA G-3′, reverse: 5′-CCA AAG TCC CGA GGT CAA-3′; *Mtor*, forward: 5′-ACC AAT TAT ACT CGC TCC CTG-3′, reverse: 5′-GTC ATA GCA ACC TCA AAG CA-3′; *Gapdh*, forward: 5′- ATG TTC CAG TAT GAC TCC ACT CAC G-3′, reverse: 5′-GAA GAC ACC AGT AGA CTC CAC GAC A-3′. All gene expression was analyzed using the comparative Ct method (2^−ΔΔCt^), where ΔΔCt = ΔCt (sample) − ΔCt (reference) relative to *Gapdh* levels.

### 2.10. Western Blot Analysis

Rat BMSCs were lysed in RIPA buffer supplemented with protease and phosphatase inhibitors. Protein extraction and electrophoresis were performed as previously described [29]. The following primary antibodies were used: AKT (#4691; 1:1000) and p-AKT (#4060; 1:2000) from Cell Signaling Technology (Danvers, MA, USA); ERK (sc-93; 1:1000) and p-ERK (sc-7383; 1:100) from Santa Cruz Biotechnology (Dallas, TX, USA); and β-actin (MAB1501; 1:10,000) from Merck Millipore (Burlington, MA, USA). After incubation with the specific primary antibody overnight at 4 °C followed by HRP-conjugated secondary antibodies for 2 h at room temperature, protein bands were visualized using an enhanced chemiluminescence system (GERPN2232, GE Healthcare, Uppsala, Sweden) and captured with a UVP ChemStudio Plus (Analytik Jena, Jena, Germany). Band intensities were quantified using Image-Pro Plus 4.5 software (Media Cybernetics, Silver Spring, MD, USA).

### 2.11. Statistical Analysis

All statistical analyses were performed using SigmaPlot version 12.5 (Systat Software Inc., San Jose, CA, USA). Data represent the mean ± standard error of the mean (SEM). For RT-qPCR analysis of mRNA levels, comparisons between two groups were performed using Student’s *t*-test. For all other data involving multiple groups, statistical significance was determined by one-way ANOVA followed by Tukey post hoc test. A *p* value < 0.05 was considered statistically significant.

## 3. Results

### 3.1. Effects of DXM on Rat BMSCs Adipogenic Differentiation

**Figure 1:**
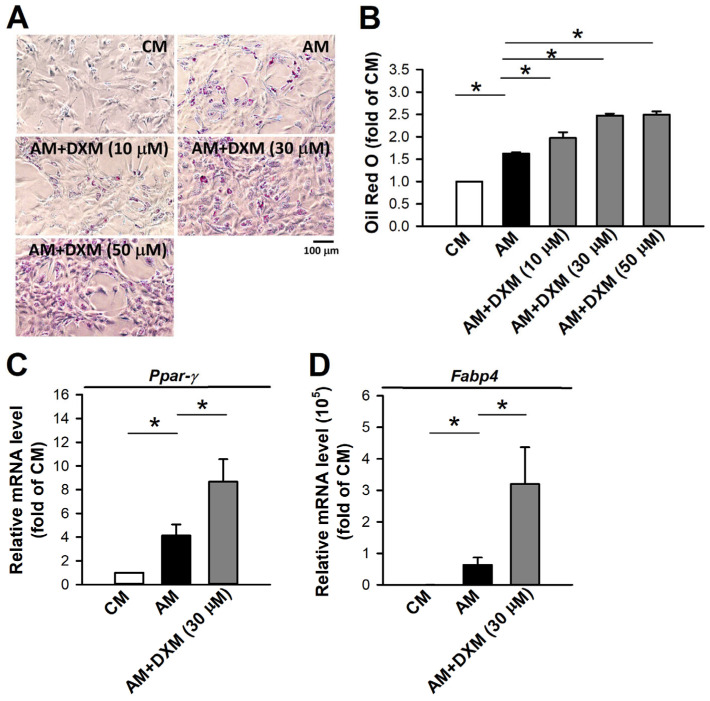
DXM enhances adipogenesis in rat BMSCs. Rat BMSCs were cultured in culture medium (CM) or adipogenic medium (AM) with or without DXM (10–50 μM) for 10–13 days. Adipogenic differentiation was assessed by Oil Red O staining (**A**), and quantification of lipid droplet accumulation showed significantly increased lipid content in AM treatment compared with CM treatment. Co-treatment with AM and DXM further increased Oil Red O staining compared with AM alone, *N* = 10 (**B**). The mRNA levels of adipogenic markers, including *Pparγ* (**C**) and *Fabp4* (**D**), were significantly increased in AM treatment compared with CM treatment and further enhanced by co-treatment with AM and DXM (30 μM) together compared with AM treatment alone, *N* = 9. Data are presented as mean ± SEM. * *p* < 0.05. CM, culture medium; AM, adipogenic medium; DXM, dextromethorphan.

To determine whether DXM modulated adipogenic differentiation, rat BMSCs were cultured in AM for 10–13 days, with CM serving as the control. CM exhibited no apparent adipogenic differentiation, whereas AM significantly induced lipid droplet formation, confirming effective adipogenic induction. Co-treatment with AM and DXM (10–50 μM) further enhanced adipogenesis compared with AM treatment alone, with progressive increases in both the cell number and staining intensity of Oil Red O-positive lipid droplets (Figure 1A). Quantification of eluted Oil Red O confirmed that AM increased lipid accumulation compared with CM treatment and that DXM produced an additional enhancement under AM conditions (Figure 1B). Consistent with these phenotypic changes, AM significantly increased the levels of *Pparγ* and *Fabp4* mRNA compared with CM treatment, and co-treatment with AM and DXM (30 μM) further elevated the mRNA levels of *Pparγ* (Figure 1C) and *Fabp4* (Figure 1D) compared with AM treatment alone. Based on these results, DXM at 30 μM was used for all subsequent experiments unless otherwise indicated. Collectively, these results indicate that DXM enhanced adipogenic differentiation of rat BMSCs, reflected by increased lipid accumulation and the upregulation of adipogenic marker genes.

### 3.2. Effects of Ca^2+^-Related and PLC–IP3R Signaling on the DXM-Enhanced Adipogenic Differentiation in Rat BMSCs

**Figure 2:**
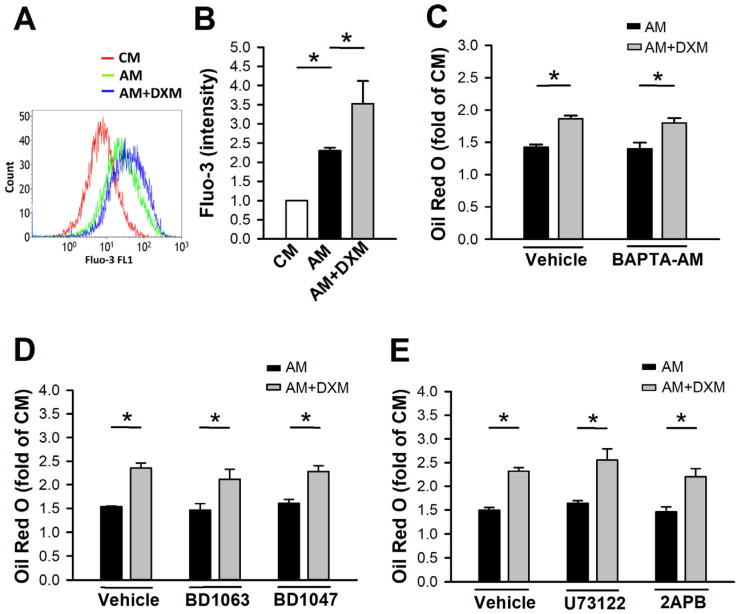
Ca^2+^ and Sig-1R/PLC–IP3 inhibitions do not attenuate DXM-induced adipogenesis in rat BMSCs. Rat BMSCs were cultured in culture medium (CM) or adipogenic medium (AM) with or without DXM (30 μM) for 10–13 days. Intracellular Ca^2+^ was assessed using Fluo-3 fluorescence. Representative histograms show the distribution of Fluo-3 fluorescence intensity (**A**), and quantification of mean fluorescence intensity demonstrated that AM treatment significantly increased Ca^2+^-related fluorescence compared with CM treatment, while co-treatment with AM and DXM further increased Ca^2+^-related fluorescence compared with AM treatment alone, *N* = 4 (**B**). Adipogenic differentiation was assessed by Oil Red O staining and quantification of lipid droplet accumulation. The DXM-increased Oil Red O staining was not significantly attenuated by co-treatment with the intracellular Ca^2+^ chelator BAPTA-AM (5 μM), *N* = 10 (**C**), Sig-1R antagonists BD1063 (10 μM) or BD1047 (1 μM), *N* = 5 (**D**), or PLC–IP3 pathway inhibitors U73122 (2 μM) or 2-APB (50 μM), *N* = 8 (**E**). Data are presented as mean ± SEM. * *p* < 0.05. CM, culture medium; AM, adipogenic medium; DXM, dextromethorphan.

To clarify whether Ca^2+^-associated modules contributed to the DXM-enhanced adipogenic differentiation, we first assessed intracellular Ca^2+^ dynamics during adipogenic induction. Flow cytometric analysis using Fluo-3 showed that AM increased intracellular Ca^2+^ levels compared with CM treatment, and co-treatment with AM and DXM showed a further upward trend (Figure 2A,B). We then tested whether intracellular Ca^2+^ availability is required for the increased pro-adipogenic effect of DXM. Chelation of intracellular Ca^2+^ with BAPTA-AM did not significantly alter the AM-induced adipogenesis and did not attenuate the increased lipid accumulation induced by DXM (Figure 2C). Because Sig-1R has been implicated in Ca^2+^ homeostasis and differentiation-related processes, we next examined the involvement of Sig-1R in DXM-enhanced adipogenic differentiation using BD1063 (10 μM) or BD1047 (1 μM). As shown in Figure 2D, both BD1063 and BD1047 did not significantly alter AM-induced adipogenesis or DXM-enhanced lipid accumulation. Finally, we blocked PLC-IP3R-associated Ca^2+^ signaling using the PLC inhibitor U73122 (2 μM) or 2-APB (50 μM, an IP3R-related Ca^2+^ signaling modulator); both interventions did not alter AM-induced adipogenesis or DXM-enhanced adipogenesis (Figure 2E). Collectively, these findings suggest that DXM-enhanced adipogenesis is maintained despite the pharmacological blockade of intracellular Ca^2+^ availability and key Ca^2+^-associated modules, including Sig-1R- and PLC-IP3R-related signaling.

### 3.3. Effects of ROS and NMDAR Signaling on the DXM-Enhanced Adipogenic Differentiation in Rat BMSCs

**Figure 3:**
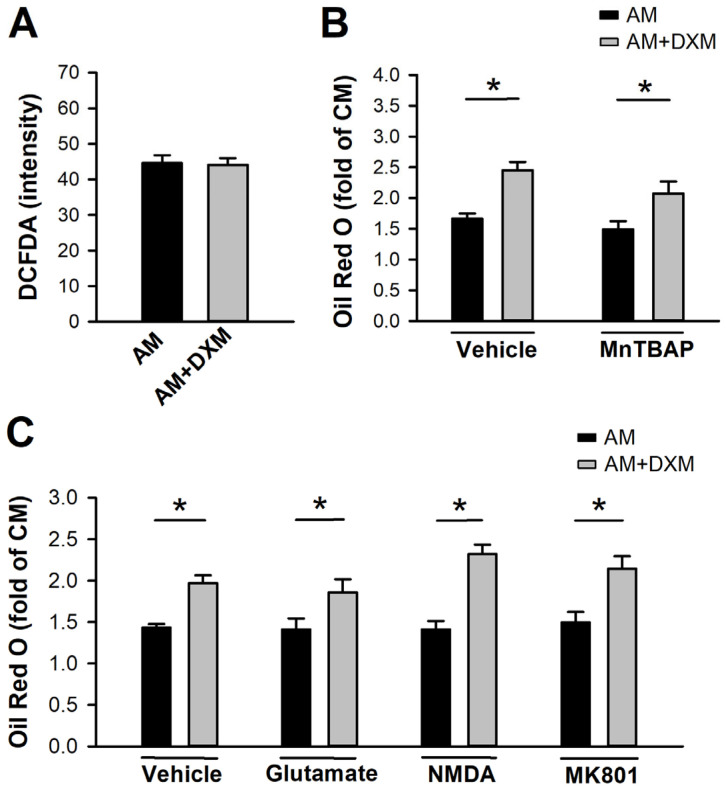
ROS scavenging and NMDAR modulation do not attenuate the DXM-induced adipogenesis in rat BMSCs. Rat BMSCs were cultured in adipogenic medium (AM) with or without DXM (30 μM) for 10–13 days. Intracellular ROS levels assessed by DCFDA fluorescence using flow cytometry were not significantly changed by DXM treatment, *N* = 5 (**A**). Adipogenic differentiation was assessed by Oil Red O staining and quantification of lipid droplet accumulation. The DXM-increased Oil Red O staining was not significantly attenuated by co-treatment with DXM and the ROS scavenger MnTBAP (10 μM), *N* = 6 (**B**), NMDAR agonists L-glutamate (30 μM) or NMDA (100 μM), or NMDAR antagonist MK-801 (10 μM), *N* = 10 (**C**). Data are presented as mean ± SEM. * *p* < 0.05. AM, adipogenic medium; DXM, dextromethorphan.

To determine whether ROS signaling contributed to the DXM-enhanced adipogenesis, intracellular ROS levels and lipid accumulation were examined under adipogenic induction. DCFDA-based flow cytometry showed that co-treatment with AM and DXM did not significantly increase intracellular ROS levels compared with AM treatment alone (Figure 3A). Consistently, ROS scavenging with MnTBAP (10 μM) neither significantly altered the AM-induced adipogenesis nor suppressed the increased lipid accumulation induced by DXM (Figure 3B), suggesting that ROS signaling was not a dominant requirement for mediating the DXM-enhanced adipogenesis under our experimental conditions. To evaluate the involvement of NMDAR DXM-induced adipogenesis, NMDAR agonists and antagonists were applied during adipogenic induction. Co-treatment with DXM and L-glutamate (30 μM) or NMDA (100 μM) did not significantly attenuate the DXM-enhanced lipid accumulation (Figure 3C). Similarly, co-treatment with DXM and the NMDAR antagonist MK-801 (10 μM) did not significantly alter adipogenesis or DXM-enhanced adipogenesis (Figure 3C). Collectively, these results suggest that DXM-enhanced adipogenesis is not predominantly mediated by ROS signaling or NMDAR-related pathways.

### 3.4. Effects of AKT/ERK Signaling, Kv Channels, and TAS2Rs on the DXM-Enhanced Adipogenic Differentiation in Rat BMSCs

**Figure 4:**
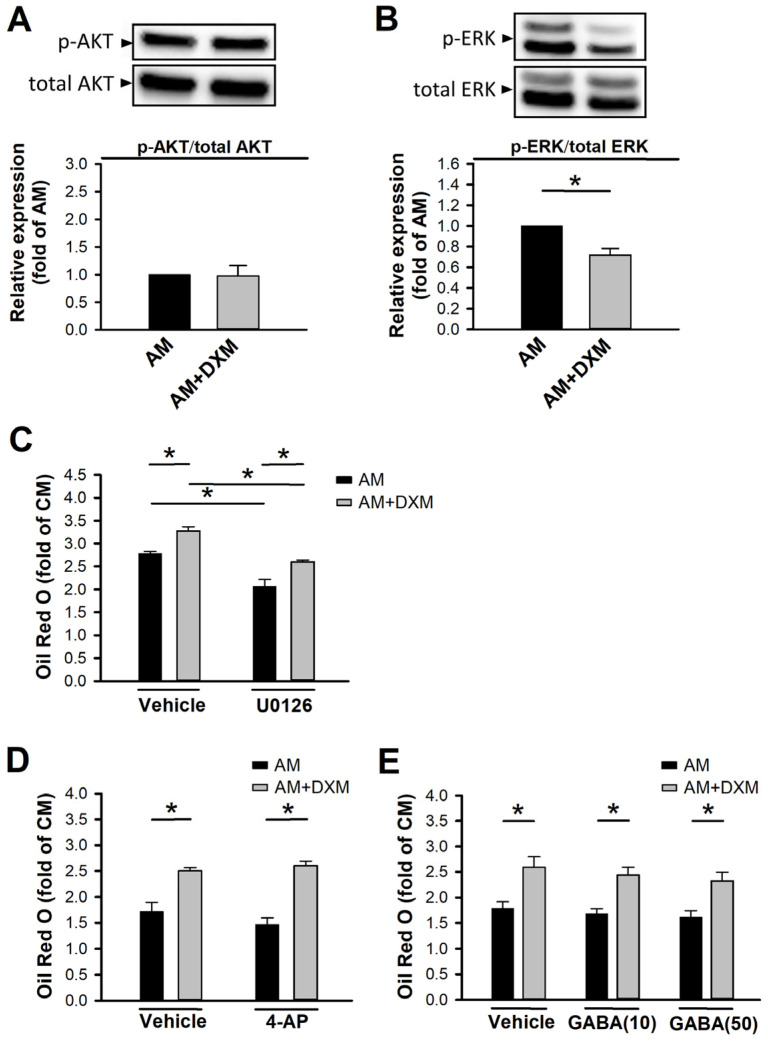
AKT/ERK, Kv channel, and TAS2R-related signaling inhibition do not attenuate DXM-induced adipogenesis in rat BMSCs. Rat BMSCs were cultured in adipogenic medium (AM) with or without DXM (30 μM) for 10–13 days. The level of AKT phosphorylation assessed by Western blotting was not significantly changed by DXM treatment, *N* = 11 (**A**). The level of ERK phosphorylation assessed by Western blotting was significantly reduced in co-treatment with AM and DXM, *N* = 11 (**B**). Adipogenic differentiation was assessed by Oil Red O staining and quantification of lipid droplet accumulation. U0126 (10 μM) reduced the AM-induced adipogenesis, yet DXM still induced an additional increase in lipid droplet accumulation in the presence of U0126, *N* = 5 (**C**), the Kv channel blocker 4-AP (1 μM), *N* = 8 (**D**), or GABA (10 μM and 50 μM), an activator of TAS2R-related signaling, *N* = 12 (**E**). Data are presented as mean ± SEM. * *p* < 0.05. AM, adipogenic medium; DXM, dextromethorphan.

To further probe signaling modules commonly implicated in adipogenesis and cell fate regulation, we evaluated AKT and ERK signaling, Kv channel activity, and TAS2Rs during adipogenic induction. Immunoblotting analysis showed that co-treatment with AM and DXM did not significantly alter AKT phosphorylation (Figure 4A), whereas it significantly reduced ERK phosphorylation compared with AM treatment alone (Figure 4B). To further assess the functional involvement of MEK/ERK signaling, cells were co-treated with the MEK inhibitor U0126 during adipogenic induction. U0126 (10 μM) reduced lipid accumulation under both basal adipogenic and DXM-treated conditions; however, DXM still produced an additional increase in lipid accumulation in the presence of U0126 (Figure 4C). These findings indicate that MEK/ERK signaling contributes to adipogenic differentiation in general, but the incremental pro-adipogenic effect of DXM is not mediated through the MEK-mediated signaling pathway. Because membrane bioelectric inputs can influence intracellular signaling states and differentiation outcomes, we next examined the effect of Kv channels on DXM-enhanced lipid accumulation using 4-AP (1 μM) to block the Kv channel. As shown in Figure 4D, blockade of the Kv channel did not significantly diminish DXM-enhanced lipid accumulation. To further examine whether TAS2R-related signaling influenced the DXM response, rat BMSCs were treated with GABA (10 and 50 μM) during adipogenic induction. As shown in Figure 4E, GABA did not significantly alter basal adipogenesis or DXM-enhanced lipid accumulation. Collectively, these findings suggest that DXM-enhanced adipogenesis is maintained despite the pharmacological inhibition of AKT/ERK signaling, Kv channel activity, and GABA-associated stimulation. Because adipogenic and osteogenic differentiation are reciprocally regulated lineage programs in MSCs, we next examined whether the DXM-enhanced adipogenic phenotype was accompanied by a reciprocal suppression of osteogenic differentiation, and tested whether DXM shifts the overall osteogenic–adipogenic balance.

### 3.5. Effects of DXM on Rat BMSCs Osteogenic Differentiation

**Figure 5:**
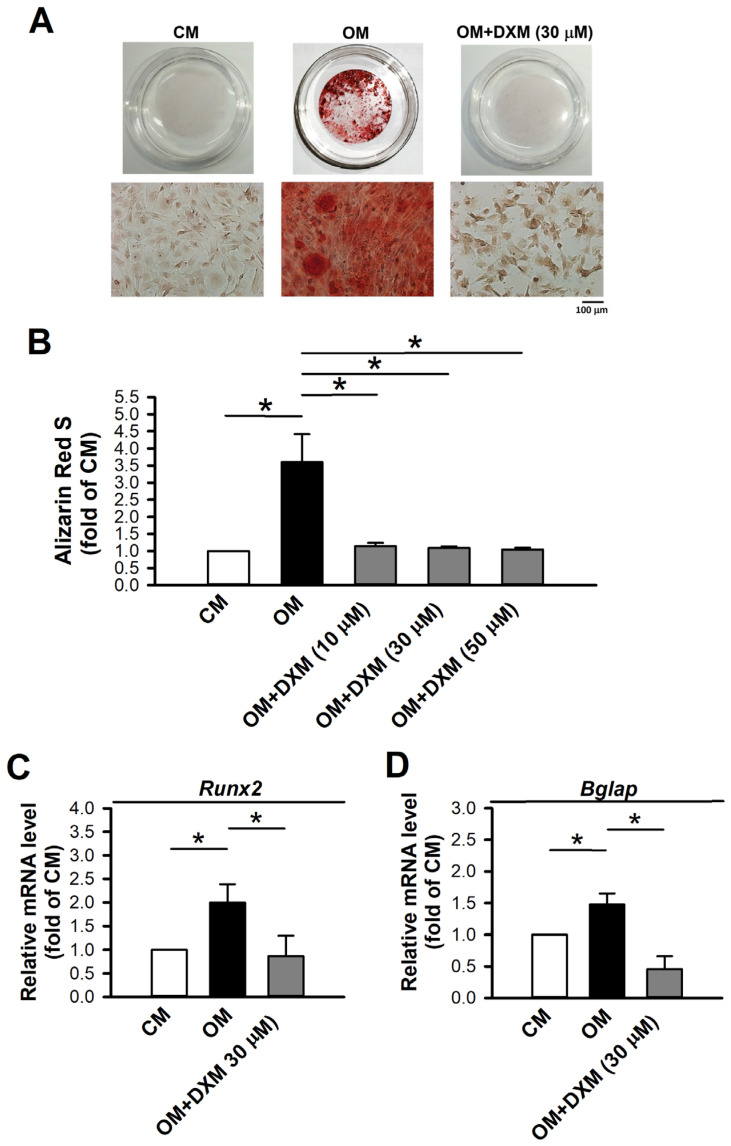
DXM suppresses osteogenesis in rat BMSCs. Rat BMSCs were cultured in culture medium (CM) or osteogenic medium (OM) with or without DXM (10–50 μM) for 21–24 days. Osteogenic differentiation was assessed by Alizarin Red S staining (**A**). Quantification of matrix mineralization showed that OM treatment significantly increased calcium deposits compared with CM treatment; co-treatment with OM and DXM abolished the OM-increased calcium deposits, *N* = 5 (**B**). The mRNA levels of osteogenic markers, including *Runx2* (**C**) and *Bglap* (**D**), were significantly increased in OM treatment compared with CM treatment, and these effects were abolished by co-treatment with OM and DXM (30 μM), *N* = 4. Data are presented as mean ± SEM. * *p* < 0.05. CM, culture medium; OM, osteogenic medium; DXM, dextromethorphan.

To evaluate the complementary osteogenic arm of this lineage-balance framework, rat BMSCs were cultured in OM for 21–24 days, with CM serving as the control. CM exhibited no apparent osteogenic differentiation, whereas OM induced prominent mineralized matrix formation, confirming effective osteogenic induction (Figure 5A). Co-treatment with OM and DXM (10–50 μM) markedly reduced mineralized matrix formation compared with OM treatment alone (Figure 5A). Quantification of eluted Alizarin Red S confirmed that OM increased mineralization relative to CM and that DXM co-treatment with OM and DXM together significantly reduced mineralization compared with OM treatment alone (Figure 5B). At the transcriptional level, OM significantly increased the levels of *Runx2* and *Bglap* mRNA compared with CM, whereas co-treatment with OM and DXM (30 μM) significantly decreased the mRNA levels of *Runx2* (Figure 5C) and *Bglap* (Figure 5D) compared with OM alone. Based on these results, DXM at 30 μM was used for all subsequent experiments unless otherwise indicated. Collectively, these results demonstrated that DXM suppressed osteogenic differentiation of rat BMSCs at both the functional level of matrix mineralization and the level of osteogenic gene expression.

### 3.6. Effects of TAZ and mTOR on the DXM-Regulated Adipogenic and Osteogenic Differentiation in Rat BMSCs

**Figure 6:**
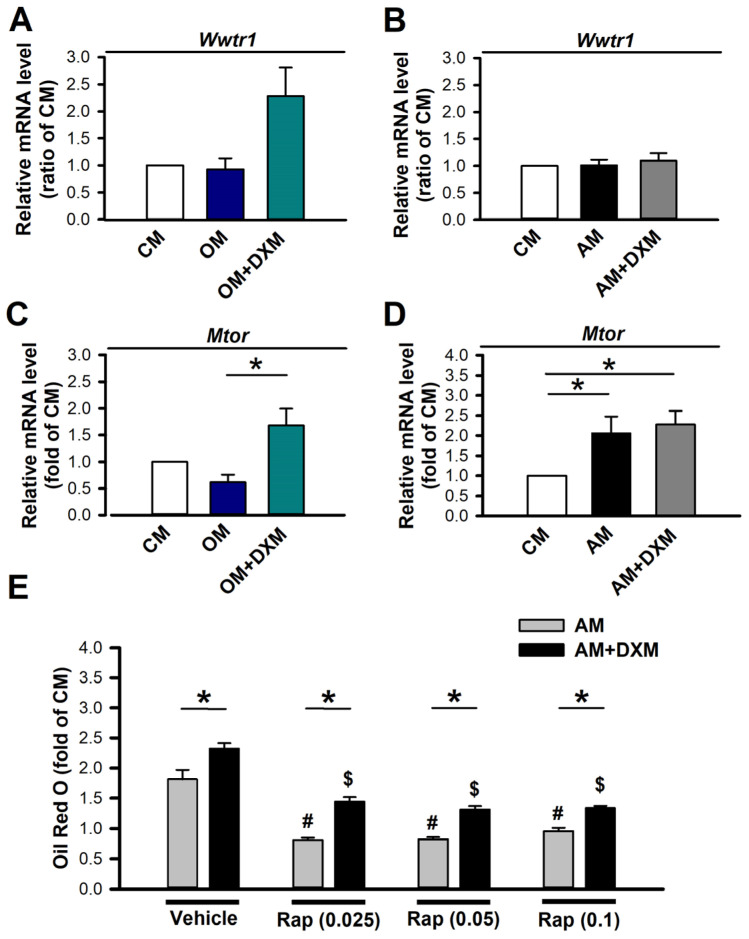
The levels of TAZ are not significantly changed by OM and AM, and mTOR inhibition does not attenuate DXM-enhanced adipogenesis in rat BMSCs. Rat BMSCs were cultured in culture medium (CM) or osteogenic medium (OM) with or without DXM (30 μM) for 21–24 days. The mRNA level of *Wwtr1* was not significantly changed by OM, *N* = 9 (**A**), or AM, *N* = 10 (**B**), treatment compared with CM treatment alone or OM and AM co-treatment with DXM compared with OM and AM alone. The mRNA level of *Mtor* was significantly decreased in OM treatment compared with CM treatment and increased by co-treatment with OM and DXM compared with OM treatment alone, *N* = 6 (**C**). The mRNA level of *Mtor* was significantly increased in AM treatment compared with CM treatment and not additionally increased by co-treatment with AM and DXM compared with AM treatment alone, *N* = 5 (**D**). Adipogenic differentiation was assessed by Oil Red O staining and quantification of lipid droplet accumulation. The DXM-increased lipid droplet accumulation was not significantly attenuated by co-treatment with the mTOR inhibitor rapamycin (0.025–0.1 μM), *N* = 5 (**E**). Data are presented as mean ± SEM. * *p* < 0.05; ^#^ *p* < 0.05, versus the vehicle (AM) group; ^$^ *p* < 0.05, versus the vehicle (AM + DXM) group. CM, culture medium; AM, adipogenic medium; OM, osteogenic medium; DXM, dextromethorphan.

To assess whether DXM modulates lineage outcomes through canonical regulatory nodes, we examined the *Wwtr1* mRNA level under adipogenic and osteogenic induction. Our results showed that DXM did not significantly alter the level of *Wwtr1* mRNA during either adipogenic (Figure 6A) or osteogenic (Figure 6B) differentiation, arguing against a major transcriptional contribution of *Wwtr1* in this model. We next examined the *Mtor* mRNA level under adipogenic and osteogenic induction with or without DXM. Under osteogenic induction, the *Mtor* mRNA level was reduced compared with CM, and co-treatment with OM and DXM together prevented OM-induced reduction (Figure 6C). Under adipogenic induction, the *Mtor* mRNA level was significantly increased compared with CM treatment and was not further increased by DXM (Figure 6D). To determine whether mTOR activity is required for DXM-enhanced adipogenesis, rapamycin was applied during adipogenic induction. Although inhibition of mTOR by rapamycin significantly reduced adipogenesis, DXM consistently enhanced lipid accumulation across all conditions. The inability of rapamycin to fully abolish the pro-adipogenic effect of DXM suggests that DXM promoted adipogenic differentiation through mTOR-independent mechanisms (Figure 6E). Collectively, these results suggest that DXM-enhanced adipogenesis is maintained despite mTOR inhibition and without detectable changes in the *Wwtr1* mRNA level.

## 4. Discussion

**Figure 7:**
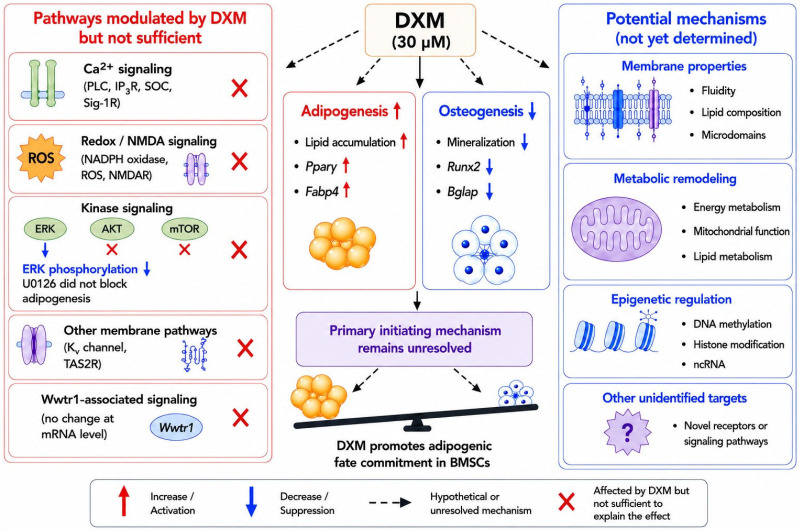
Proposed model summarizing the effects of DXM on rat BMSC differentiation and the mechanistic pathways evaluated in the present study. DXM (30 μM) promoted adipogenic differentiation, as evidenced by increased lipid accumulation and the upregulation of adipogenic markers including *Pparγ* and *Fabp4*, while simultaneously suppressing osteogenic differentiation, as evidenced by reduced mineralization and decreased expression of osteogenic markers such as *Runx2* and *Bglap*. Overall, DXM shifted the differentiation balance toward adipogenesis and away from osteogenesis. Multiple canonical signaling pathways potentially associated with DXM action were experimentally evaluated, including Ca^2+^-related signaling pathways (PLC, IP_3_R, SOC channels, and Sig-1R), redox/NMDA-related signaling, kinase signaling pathways (ERK, AKT, and mTOR), other membrane-associated pathways (K_v_ channels and TAS2R), and osteogenic transcriptional regulators including *Wwtr1*. Although several pathways were modulated by DXM, none were sufficient to fully explain the pro-adipogenic effect of DXM. In particular, DXM reduced ERK phosphorylation; however, pharmacological inhibition of ERK signaling with U0126 did not alter DXM-induced adipogenesis, suggesting that ERK modulation alone is not functionally required for the observed phenotype. Because the initiating mechanism underlying DXM-induced adipogenic bias remains unresolved, several potential mechanisms are proposed for future investigation, including alterations in membrane properties, metabolic remodeling, epigenetic regulation, and other unidentified signaling targets. Dashed arrows indicate hypothetical or incompletely defined mechanisms. Red upward arrows indicate increased activity or expression, whereas blue downward arrows indicate suppression or reduction. Red crosses denote pathways that were evaluated but were insufficient to fully explain DXM-induced adipogenic differentiation.

The present study identified DXM as a modulator of mesenchymal stem cell differentiation in rat BMSCs, enhancing adipogenic differentiation while suppressing osteogenic differentiation under lineage-inducing conditions. DXM increased lipid droplet accumulation during adipogenic induction and reduced matrix mineralization during osteogenic induction, accompanied by concordant shifts in lineage-associated gene expression, including increased adipogenic markers (*Pparγ* and *Fabp4*) and decreased osteogenic markers (*Runx2* and *Bglap*). Notably, systematic pharmacological and transcriptional interrogation across multiple candidate modules demonstrated that the incremental pro-adipogenic effect of DXM was not significantly attenuated under the conditions tested, including Ca^2+^-associated pathways (including Sig-1R- and PLC-IP3R-related signaling), ROS signaling, NMDAR-related inputs, AKT/ERK cascades, Kv channel activity, TAS2R-related signaling, or *Wwtr1* and *Mtor*-associated regulation under our experimental conditions (Figure 7).

Calcium signaling has been widely reported to participate in adipocyte differentiation, acting through intracellular Ca^2+^ dynamics and downstream effectors such as the PLC-IP3R cascade [30,31]. Mechanistically, PLC-IP3R signaling is commonly considered as a canonical route linking membrane cues to intracellular Ca^2+^ mobilization, in which PLC-mediated IP3 production triggers IP3R-dependent Ca^2+^ release from the endoplasmic reticulum (ER) [32,33]. In parallel, Sig-1R is positioned at the ER, including ER–mitochondria contact sites, where it can modulate Ca^2+^ signaling by influencing IP3R stability and ER-to-mitochondria Ca^2+^ transfer, and has been implicated in Ca^2+^ homeostasis and differentiation-related processes [19,34]. Although adipogenic induction increased intracellular Ca^2+^ levels, inhibiting intracellular Ca^2+^ availability, Sig-1R- and PLC-IP3R-related signaling did not significantly attenuate DXM-enhanced adipogenesis. Thus, DXM-associated Ca^2+^ changes appear to accompany adipogenic induction but are not sufficient to explain the additional pro-adipogenic effect of DXM when Ca^2+^-associated modules are pharmacologically suppressed.

ROS signaling and NOX activity have been implicated in adipogenic differentiation, with moderate ROS acting as second messengers that shape lineage-associated transcriptional programs [35,36]. Mechanistically, NOX complexes generate superoxide and downstream ROS in a regulated manner, and receptor-linked Ca^2+^ inputs can promote NOX activation through Ca^2+^-dependent kinases and small GTPases, coupling extracellular cues to redox signaling [37,38]. Because NMDAR activation increases intracellular Ca^2+^ and has been linked in several systems to NOX-dependent ROS production, an NMDAR-Ca^2+^-NOX-ROS axis has been proposed as a feed-forward redox circuit [39,40]. Given prior reports that DXM can modulate both NOX-linked oxidative stress and NMDAR-related signaling [7,9], we evaluated whether this redox circuit contributed to DXM-enhanced adipogenesis. Our results showed that co-treatment with AM and DXM did not significantly increase DCFDA-detected intracellular ROS, MnTBAP did not significantly attenuate DXM-enhanced lipid accumulation, and NMDAR agonists or MK-801 did not significantly alter the DXM pro-adipogenic phenotype. Together, these findings argue against a dominant requirement for an NMDAR-linked NOX-ROS loop in mediating DXM-enhanced adipogenesis under our experimental conditions.

Several kinase-dependent signaling pathways, including PI3K-AKT, MEK-ERK, and mTOR, are well-established regulators of mesenchymal stem cell differentiation and metabolic control [5,14]. mTOR, in particular, has been implicated as a nutrient-sensing node that can influence mesenchymal lineage specification and adipogenic capacity [5,41]. In addition to kinase cascades, membrane bioelectric inputs such as Kv channels can modulate intracellular signaling states and differentiation outcomes in a context-dependent manner [24,25]. TAS2Rs can engage second-messenger pathways, commonly via PLC-dependent transduction, that intersect with broader signaling networks controlling cell behavior [21,26]. Moreover, the transcriptional co-activator TAZ is a recognized rheostat for adipogenic–osteogenic balance [27]. In our study, DXM reduced ERK phosphorylation accompanied by changes in *Mtor* transcript levels during differentiation. Functional pharmacological tests further showed that U0126 reduced lipid accumulation under both basal adipogenic and DXM-treated conditions, indicating that the MEK/ERK signaling contributes to adipogenic differentiation in general. However, DXM still produced an additional increase in lipid accumulation in the presence of U0126, suggesting that the incremental pro-adipogenic effect of DXM is not mediated by the MEK-signaling pathway. Similarly, rapamycin treatment reduced baseline adipogenesis but did not abolish the additional lipid accumulation induced by DXM. Likewise, blockade of the Kv channel with 4-AP or treatment with a TAS2R-related signaling input did not significantly attenuate DXM-enhanced adipogenesis, and *Wwtr1* mRNA levels did not significantly change. Together, these findings suggest that the DXM-driven adipogenic bias persisted under multiple perturbations of canonical kinase and membrane-associated modules. In this context, the observed decrease in p-ERK is more consistent with a parallel or secondary signaling change associated with differentiation status or cellular adaptation rather than the principal upstream driver of the DXM response.

Because multiple upstream signaling modules did not account for the DXM-driven adipogenic bias, we revisited lineage-defining transcriptional programs. PPARγ is a master regulator of adipogenesis [3], whereas Runx2 is essential for osteoblast differentiation and bone formation [4]. DXM significantly increased *Pparγ* and *Fabp4* mRNA levels while concomitantly reducing *Runx2* and *Bglap* mRNA levels, consistent with activation of an adipogenic transcriptional program coupled to suppression of osteogenic gene networks. Prior studies support reciprocal antagonism between adipogenic and osteogenic programs [42,43], and mechanistic work showed that PPARγ activation can suppress Runx2 expression and inhibit Runx2-driven osteocalcin transcription, in part through the suppression of Runx2 promoter activity and physical interaction with Runx2 [44]. Consistent with this cross-regulatory model in vivo, PPARγ insufficiency increases bone mass by enhancing osteoblastogenesis from bone marrow progenitors [45]. Thus, our expression profile is compatible with a model in which DXM promotes adipogenic programming that secondarily constrains osteogenic output, although the upstream trigger linking DXM to this transcriptional shift remains to be defined.

In this study, DXM was examined over a 10–50 μM range to capture a clear concentration–response profile in rat BMSCs, and 30 μM was selected for subsequent pathway interrogation to provide a stable, quantifiable phenotype across experimental conditions. This concentration was selected based on the observed concentration–response profile to enable reproducible phenotypic quantification and mechanistic interrogation across conditions. From a pharmacokinetic standpoint, parent DXM exposure in vivo is strongly shaped by extensive first-pass metabolism via CYP2D6, such that circulating concentrations are generally low after routine dosing but can increase substantially in poor metabolizers or in the presence of CYP2D6 inhibition [46]. In humans, quinidine-mediated CYP2D6 inhibition and the CYP2D6 phenotype have been shown to markedly alter DXM exposure and disposition, supporting the view that systemic parent-drug levels vary widely across metabolic backgrounds and drug–drug interaction settings [47,48,49,50]. In addition, bupropion has been reported to inhibit CYP2D6 activity using DXM as a probe substrate, further underscoring the potential for clinically relevant pharmacokinetic modulation [51]. Collectively, these clinical pharmacokinetic data suggest that systemic parent-drug exposure is typically well below the micromolar range with routine dosing, but can shift upward under specific metabolic and drug–drug interaction settings; therefore, whether 30 μM is achievable within the bone marrow microenvironment remains uncertain. At present, however, DXM exposure within the bone marrow niche has not been established, and local tissue levels may not mirror plasma levels because of tissue distribution and metabolic heterogeneity [46]. These considerations frame our data as demonstrating a micromolar-range in vitro capacity of DXM to bias BMSC lineage outcomes; defining marrow-relevant exposure–response relationships, particularly at lower concentrations and with direct measurement of tissue/bone marrow exposure where feasible, will further strengthen translational interpretation. Beyond systemic pharmacokinetics, the effective exposure and phenotypic output in vitro may also be influenced by the bioactive components present in the differentiation induction media (e.g., dexamethasone, insulin, and indomethacin), and potential interactions with DXM cannot be fully excluded. Complementary time-course assessments of cellular health during differentiation, together with simplified induction conditions, may help refine concentration selection and clarify whether DXM primarily amplifies specific cocktail inputs or engages an upstream driver independent of individual components.

A limitation of the present study is that the mechanism was primarily inferred from systematic pharmacological interrogation; therefore, the initiating “trigger” upstream of the DXM-driven transcriptional shift remains to be defined. Notably, DXM is not classically recognized as a PPARγ ligand; however, the observed upregulation of Pparγ mRNA under our experimental conditions is consistent with engagement of an adipogenic transcriptional program and raises the possibility that DXM influences PPARγ activity indirectly through upstream regulatory processes rather than acting as a canonical receptor agonist. Nevertheless, the persistence of the DXM pro-adipogenic phenotype across multiple perturbations provides mechanistic positioning by indicating that the effect is not readily explained by the canonical modules tested, thereby narrowing plausible upstream drivers. With respect to rapamycin, our findings should be interpreted as showing that the DXM-enhanced adipogenesis was maintained under rapamycin treatment, rather than as definitive evidence of complete mTOR independence, because canonical mTOR downstream targets such as p-p70S6K, p-S6, or p-4EBP1 were not directly assessed in this study. Future studies assessing these canonical mTOR downstream readouts will be important to clarify the extent to which rapamycin-sensitive mTOR signaling contributes to the DXM response in rat BMSCs. A practical and testable next step is to determine whether the active species in culture is DXM itself or bioactive metabolite(s) by quantifying DXM/metabolites in conditioned media and/or using metabolic enzyme inhibitors (e.g., CYP2D6 inhibitors) to distinguish parent compound- versus metabolite-driven effects.

In addition, given the unchanged *Wwtr1* mRNA level, future work should consider alternative sensing nodes (other co-regulators/chromatin-associated factors) and non-classical directions highlighted by recent studies, including membrane organization/fluidity, autophagy-linked regulation, and mitochondrial/metabolic state [52,53]. Recent reviews in bone-related fields highlight that lipid-bilayer composition and membrane biophysical properties are tunable and can shape downstream cellular responses, underscoring the broader principle that membrane organization may influence lineage outcomes. Consistent with this concept, these reviews discussed extracellular vesicles as phospholipid/lipid-bilayer systems that can be functionally “re-tuned” by manipulating membrane-associated properties and cargo loading (e.g., engineering vesicles via parental-cell modification or post-isolation modification; displaying targeting/therapeutic proteins on the membrane; and loading miRNAs/siRNAs/drugs), underscoring how membrane composition and biophysical organization can shape downstream cellular responses. Accordingly, one plausible non-classical direction is that DXM alters membrane organization (and potentially membrane fluidity or microdomain behavior) to bias how multiple receptors/channels and second-messenger systems are coordinated, without requiring a single dominant canonical cascade. Autophagy-related regulation is also highlighted as an upstream determinant of bone-cell function; for example, adipose-derived stem cell EVs have been reported to influence osteoblast-related outcomes through pathways that include autophagy (alongside MAPK and Rap-1 signaling), supporting autophagy–lysosome and autophagy–lipid droplet crosstalk as biologically plausible “trigger-level” candidates for a DXM-driven adipogenic bias. Finally, these contemporary frameworks further motivate direct testing of mitochondrial and metabolic rewiring (e.g., mitochondrial function, NAD^+^/acetyl-CoA availability, lipid-handling states) because the organelle metabolic state can act as an upstream integrator of differentiation cues and may reshape lineage-associated transcription without overt dependence on the canonical pathways interrogated here. Moreover, verifiable tests that directly probe necessity and target engagement (e.g., PPARγ antagonism or genetic loss of function, ligand-binding/PPARγ reporter assays, and unbiased lipidomics/metabolomics/epigenomic profiling) will be important to identify the upstream driver(s) of this pathway-robust lineage bias and to evaluate translational relevance in human MSCs and in vivo models.

## 5. Conclusions

This study demonstrated that DXM promoted adipogenic differentiation and suppressed osteogenic differentiation in rat BMSCs, accompanied by increased *Pparg*/*Fabp4* mRNA levels and decreased *Runx2*/*Bglap* mRNA levels. The pro-adipogenic effect of DXM was maintained despite systematic pharmacological and transcriptional interrogation of multiple canonical modules, including Ca^2+^-associated signaling, ROS, NMDAR-related inputs, TAS2R-related signaling input, AKT/ERK, and *Mtor* and *Wwtr1* mRNA expression. These findings support a non-canonical mechanism by which DXM modulated the osteogenic–adipogenic balance toward adipogenesis under our experimental conditions.

## Data Availability

The raw data supporting the conclusions of this article will be made available by the authors on request.

## References

[1] Justesen J., Stenderup K., Ebbesen E.N., Mosekilde L., Steiniche T., Kassem M. (2001). Adipocyte tissue volume in bone marrow is increased with aging and in patients with osteoporosis. Biogerontology.

[2] Devlin M.J., Rosen C.J. (2015). The bone–fat interface: Basic and clinical implications of marrow adiposity. Lancet Diabetes Endocrinol..

[3] Tontonoz P., Hu E., Graves R.A., Budavari A.I., Spiegelman B.M. (1994). Stimulation of adipogenesis in fibroblasts by PPARγ2, a lipid-activated transcription factor. Cell.

[4] Komori T., Yagi H., Nomura S., Yamaguchi A., Sasaki K., Deguchi K., Shimizu Y., Bronson R.T., Gao Y.H., Inada M. (1997). Targeted disruption of Cbfa1 results in a complete lack of bone formation owing to maturational arrest of osteoblasts. Cell.

[5] Chen Q., Shou P., Zheng C., Jiang M., Cao G., Yang Q., Cao J., Xie N., Velletri T., Zhang X. (2016). Fate decision of mesenchymal stem cells: Adipocytes or osteoblasts?. Cell Death Differ..

[6] Han L., Wang B., Wang R., Gong S., Chen G., Xu W. (2019). The shift in the balance between osteoblastogenesis and adipogenesis of mesenchymal stem cells mediated by glucocorticoid receptor. Stem Cell Res. Ther..

[7] Zhang W., Wang T., Qin L., Gao H.-M., Wilson B., Ali S.F., Zhang W., Hong J.-S., Liu B. (2004). Neuroprotective effect of dextromethorphan in the MPTP Parkinson’s disease model: Role of NADPH oxidase. FASEB J..

[8] Carpenter C.L., Marks S.S., Watson D.L., Greenberg D.A. (1988). Dextromethorphan and dextrorphan as calcium channel antagonists. Brain Res..

[9] Wong B.Y., Coulter D.A., Choi D.W., Prince D.A. (1988). Dextrorphan and dextromethorphan, common antitussives, are antiepileptic and antagonize N-methyl-D-aspartate in brain slices. Neurosci. Lett..

[10] Werling L.L., Lauterbach E.C., Calef U. (2007). Dextromethorphan as a potential neuroprotective agent with unique mechanisms of action. Neurologist.

[11] Upadhyaya J.D., Singh N., Sikarwar A.S., Chakraborty R., Pydi S.P., Bhullar R.P., Dakshinamurti S., Chelikani P. (2014). Dextromethorphan mediated bitter taste receptor activation in the pulmonary circuit causes vasoconstriction. PLoS ONE.

[12] Lai Y.-C., Yao Z.-K., Chang T.-C., Feng C.-W., Kuo T.-J., Luo Y.-W., Jean Y.-H., Lin H.Y.-H., Wen Z.-H. (2025). Dextromethorphan inhibits osteoblast differentiation and bone regeneration of rats with subcritical-sized calvarial defects. Environ. Toxicol..

[13] Jung T.W., Hwang E.J., Pyun D.H., Kim T.J., Lee H.J., Abd El-Aty A.M., Bang J.S., Kim H.-C., Jeong J.H. (2021). 3-hydroxymorphinan enhances mitochondrial biogenesis and adipocyte browning through AMPK-dependent pathway. Biochem. Biophys. Res. Commun..

[14] James A.W. (2013). Review of signaling pathways governing MSC osteogenic and adipogenic differentiation. Scientifica.

[15] Atashi F., Modarressi A., Pepper M.S. (2015). The role of reactive oxygen species in mesenchymal stem cell adipogenic and osteogenic differentiation: A review. Stem Cells Dev..

[16] Aquino-Martínez R., Artigas N., Gámez B., Rosa J.L., Ventura F. (2017). Extracellular calcium promotes bone formation from bone marrow mesenchymal stem cells by amplifying BMP-2 effects on SMAD signalling. PLoS ONE.

[17] Salasznyk R.M., Klees R.F., Hughlock M.K., Plopper G.E. (2005). ERK signaling pathways regulate the osteogenic differentiation of human mesenchymal stem cells on collagen I and vitronectin. Cell Commun. Adhes..

[18] Baker N., Sohn J., Tuan R.S. (2015). Promotion of human mesenchymal stem cell osteogenesis by PI3-kinase/Akt signaling, and the influence of caveolin-1/cholesterol homeostasis. Stem Cell Res. Ther..

[19] Hayashi T., Su T.-P. (2007). Sigma-1 receptor chaperones at the ER-mitochondrion interface regulate Ca(2+) signaling and cell survival. Cell.

[20] Chakraborty A., Murphy S., Coleman N. (2017). The Role of NMDA Receptors in Neural Stem Cell Proliferation and Differentiation. Stem Cells Dev..

[21] Tuzim K., Korolczuk A. (2021). An update on extra-oral bitter taste receptors. J. Transl. Med..

[22] Ramazzotti G., Fiume R., Chiarini F., Campana G., Ratti S., Billi A.M., Manzoli L., Follo M.Y., Suh P.-G., McCubrey J. (2019). Phospholipase C-β1 interacts with cyclin E in adipose-derived stem cells osteogenic differentiation. Adv. Biol. Regul..

[23] Bill C.A., Vines C.M. (2020). Phospholipase C. Adv. Exp. Med. Biol..

[24] Steward A.J., Kelly D.J. (2015). Mechanical regulation of mesenchymal stem cell differentiation. J. Anat..

[25] Pchelintseva E., Djamgoz M.B.A. (2018). Mesenchymal stem cell differentiation: Control by calcium-activated potassium channels. J. Cell. Physiol..

[26] Zhang Y., Hoon M.A., Chandrashekar J., Mueller K.L., Cook B., Wu D., Zuker C.S., Ryba N.J.P. (2003). Coding of Sweet, Bitter, and Umami Tastes: Different Receptor Cells Sharing Similar Signaling Pathways. Cell.

[27] Hong J.H., Hwang E.S., McManus M.T., Amsterdam A., Tian Y., Kalmukova R., Mueller E., Benjamin T., Spiegelman B.M., Sharp P.A. (2005). TAZ, a transcriptional modulator of mesenchymal stem cell differentiation. Science.

[28] Lin J.-H., Ting P.-C., Lee W.-S., Chiu H.-W., Chien C.-A., Liu C.-H., Sun L.-Y., Yang K.-T. (2019). Palmitic Acid Methyl Ester Induces G2/M Arrest in Human Bone Marrow-Derived Mesenchymal Stem Cells via the p53/p21 Pathway. Stem Cells Int..

[29] Lin J.-H., Chang H.-H., Lee W.-S., Ting P.-C., Luo Y.-P., Yang K.-T. (2021). Palmitic Acid Methyl Ester Enhances Adipogenic Differentiation in Rat Adipose Tissue-Derived Mesenchymal Stem Cells through a G Protein-Coupled Receptor-Mediated Pathway. Stem Cells Int..

[30] Shi H., Halvorsen Y.D., Ellis P.N., Wilkison W.O., Zemel M.B. (2000). Role of intracellular calcium in human adipocyte differentiation. Physiol. Genom..

[31] Neal J.W., Clipstone N.A. (2002). Calcineurin mediates the calcium-dependent inhibition of adipocyte differentiation in 3T3-L1 cells. J. Biol. Chem..

[32] Berridge M.J. (2016). The Inositol Trisphosphate/Calcium Signaling Pathway in Health and Disease. Physiol. Rev..

[33] Berridge M.J. (2009). Inositol trisphosphate and calcium signalling mechanisms. Biochim. Biophys. Acta.

[34] Su T.-P., Hayashi T., Maurice T., Buch S., Ruoho A.E. (2010). The sigma-1 receptor chaperone as an inter-organelle signaling modulator. Trends Pharmacol. Sci..

[35] Lee H., Lee Y.J., Choi H., Ko E.H., Kim J.-W. (2009). Reactive oxygen species facilitate adipocyte differentiation by accelerating mitotic clonal expansion. J. Biol. Chem..

[36] Kanda Y., Hinata T., Kang S.W., Watanabe Y. (2011). Reactive oxygen species mediate adipocyte differentiation in mesenchymal stem cells. Life Sci..

[37] Bedard K., Krause K.-H. (2007). The NOX family of ROS-generating NADPH oxidases: Physiology and pathophysiology. Physiol. Rev..

[38] Hordijk P.L. (2006). Regulation of NADPH oxidases: The role of Rac proteins. Circ. Res..

[39] Shelat P.B., Chalimoniuk M., Wang J.-H., Strosznajder J.B., Lee J.C., Sun A.Y., Simonyi A., Sun G.Y. (2008). Amyloid beta peptide and NMDA induce ROS from NADPH oxidase and AA release from cytosolic phospholipase A2 in cortical neurons. J. Neurochem..

[40] Girouard H., Wang G., Gallo E.F., Anrather J., Zhou P., Pickel V.M., Iadecola C. (2009). NMDA receptor activation increases free radical production through nitric oxide and NOX2. J. Neurosci..

[41] Xiang X., Zhao J., Xu G., Li Y., Zhang W. (2011). mTOR and the differentiation of mesenchymal stem cells. Acta Biochim. Biophys. Sin. (Shanghai).

[42] Lecka-Czernik B., Moerman E.J., Grant D.F., Lehmann J.M., Manolagas S.C., Jilka R.L. (2002). Divergent effects of selective peroxisome proliferator-activated receptor-γ2 ligands on adipocyte versus osteoblast differentiation. Endocrinology.

[43] Moerman E.J., Teng K., Lipschitz D.A., Lecka-Czernik B. (2004). Aging activates adipogenic and suppresses osteogenic programs in mesenchymal marrow stroma/stem cells: The role of PPAR-gamma2 transcription factor and TGF-beta/BMP signaling pathways. Aging Cell.

[44] Jeon M.J., Kim J.A., Kwon S.H., Kim S.W., Park K.S., Park S.W., Kim S.Y., Shin C.S. (2003). Activation of peroxisome proliferator-activated receptor-γ inhibits the Runx2-mediated transcription of osteocalcin in osteoblasts. J. Biol. Chem..

[45] Akune T., Ohba S., Kamekura S., Yamaguchi M., Chung U.I., Kubota N., Terauchi Y., Harada Y., Azuma Y., Nakamura K. (2004). PPARγ insufficiency enhances osteogenesis through osteoblast formation from bone marrow progenitors. J. Clin. Investig..

[46] Taylor C.P., Traynelis S.F., Siffert J., Pope L.E., Matsumoto R.R. (2016). Pharmacology of dextromethorphan: Relevance to dextromethorphan/quinidine (Nuedexta®) clinical use. Pharmacol. Ther..

[47] Capon D.A., Bochner F., Kerry N., Mikus G., Danz C., Somogyi A.A. (1996). The influence of CYP2D6 polymorphism and quinidine on the disposition and antitussive effect of dextromethorphan in humans. Clin. Pharmacol. Ther..

[48] Schadel M., Wu D., Otton S.V., Kalow W., Sellers E.M. (1995). Pharmacokinetics of dextromethorphan and metabolites in humans: Influence of the CYP2D6 phenotype and quinidine inhibition. J. Clin. Psychopharmacol..

[49] Pope L.E., Khalil M.H., Berg J.E., Stiles M., Yakatan G.J., Sellers E.M. (2004). Pharmacokinetics of dextromethorphan after single or multiple dosing in combination with quinidine in extensive and poor metabolizers. J. Clin. Pharmacol..

[50] Zhang Y., Britto M.R., Valderhaug K.L., Wedlund P.J., Smith R.A. (1992). Dextromethorphan: Enhancing its systemic availability by way of low-dose quinidine-mediated inhibition of cytochrome P4502D6. Clin. Pharmacol. Ther..

[51] Kotlyar M., Brauer L.H., Tracy T.S., Hatsukami D.K., Harris J., Bronars C.A., Adson D.E. (2005). Inhibition of CYP2D6 activity by bupropion. J. Clin. Psychopharmacol..

[52] Wu Y., Song P., Wang M., Liu H., Jing Y., Su J. (2024). Extracellular derivatives for bone metabolism. J. Adv. Res..

[53] Liu H., Li R., Yang H., Situ B., Wang G., Xu K., Su J. (2025). Extracellular vesicles in gut-bone axis: Novel insights and therapeutic opportunities for osteoporosis. Small Sci..

